# Modeling of the Point Defect Migration across the AlN/GaN Interfaces—Ab Initio Study

**DOI:** 10.3390/ma15020478

**Published:** 2022-01-09

**Authors:** Roman Hrytsak, Pawel Kempisty, Ewa Grzanka, Michal Leszczynski, Malgorzata Sznajder

**Affiliations:** 1 Institute of High Pressure Physics, Polish Academy of Sciences, Sokolowska 29/37, 01-142 Warsaw, Poland; rhrytsak@unipress.waw.pl (R.H.); Pawel.Kempisty@unipress.waw.pl (P.K.); elesk@unipress.waw.pl (E.G.); mike@unipress.waw.pl (M.L.); 2 Institute of Physics, College of Natural Sciences, University of Rzeszow, Pigonia 1, 35-959 Rzeszow, Poland

**Keywords:** density functional theory, nitrides, interfaces, heterostructures, point defect diffusion, diffusion across the interface

## Abstract

The formation and diffusion of point defects have a detrimental impact on the functionality of devices in which a high quality AlN/GaN heterointerface is required. The present paper demonstrated the heights of the migration energy barriers of native point defects throughout the AlN/GaN heterointerface, as well as the corresponding profiles of energy bands calculated by means of density functional theory. Both neutral and charged nitrogen, gallium, and aluminium vacancies were studied, as well as their complexes with a substitutional III-group element. Three diffusion mechanisms, that is, the vacancy mediated, direct interstitial, and indirect ones, in bulk AlN and GaN crystals, as well at the AlN/GaN heterointerface, were taken into account. We showed that metal vacancies migrated across the AlN/GaN interface, overcoming a lower potential barrier than that of the nitrogen vacancy. Additionally, we demonstrated the effect of the inversion of the electric field in the presence of charged point defects $V_{Ga}^{3-}$ and $V_{Al}^{3-}$ at the AlN/GaN heterointerface, not reported so far. Our findings contributed to the issues of structure design, quality control, and improvement of the interfacial abruptness of the AlN/GaN heterostructures.

## 1. Introduction

III–V nitride materials, in particular GaN and AlN, found many interesting applications in high-power, high-temperature electronic and optoelectronic devices, such as laser diodes (LDs), light emitting diodes (LEDs), near-ultraviolet distributed Bragg reflectors (DBRs), transparent biosensors, or plasma-wave terahertz emitters [1,2,3,4,5]. Due to a wide direct band gap lying in the range from 3.47 eV (GaN) to 6.09 eV (AlN) [6,7,8], the ${\mathrm{Al}}_{x}{\mathrm{Ga}}_{1-x}N$ alloy system also enabled the fabrication of photodetectors and high electron mobility transistors (HEMTs) [9,10,11]. The main component of the active layers in such device structures are the AlGaN/GaN heterostructures whose interfacial quality is crucial for achieving a high performance of the AlGaN-based devices. The AlN/GaN interfacial quality can be degraded by a number of factors including a high density of threading dislocations, stacking faults, interface roughness, point defects, or cracking highly strained AlN layer. Besides, AlGaN/GaN HEMTs can operate in a harsh environment where a high-energy particle radiation can be present. Under such harsh conditions the reliability, performance and life-time of the device can be influenced by the presence of defects in the structure and their diffusion.

Experimental studies on the Al diffusion in GaN during metal organic vapor phase epitaxy (MOVPE) or metal organic chemical vapor deposition (MOCVD) of AlGaN/GaN and AlN/GaN structures were investigated recently in [12,13]. In particular, Chaaben et al. investigated the Al diffusion from AlN interlayer to GaN in a sandwiched structure made of alternating GaN/AlN/GaN and capped with AlN layer. The reported in- and out- Al diffusion from AlN interlayer to GaN layers and from AlN cap layers to GaN were found to be around $10^{-14}$${\mathrm{cm}}^{2}$$s^{-1}$. Additionally, the reported Al diffusion at the AlN/GaN interface was about one order of magnitude higher than the one at ${\mathrm{Al}}_{x}{\mathrm{Ga}}_{1-x}N$/GaN (x<4%) interface [13]. Kim et al. [14] investigated in turn the electrical and interfacial properties of AlN/GaN heterostructures with different dielectric protection layers such as ${\mathrm{Al}}_{2}O_{3}$, ${\mathrm{HfO}}_{2}$, and ${\mathrm{HfO}}_{2}/{\mathrm{Al}}_{2}O_{3}$. The interface trap density ($D_{it}$) obtained from C–V plots using Therman method revealed certain peaks at ∼0.25 eV for the ${\mathrm{Al}}_{2}O_{3}/\mathrm{AlN}$ and ${\mathrm{HfO}}_{2}/{\mathrm{Al}}_{2}O_{3}/\mathrm{AlN}$ samples, as well as at ∼0.52 eV for the sample with ${\mathrm{HfO}}_{2}$, associated with nitrogen vacancy-related defects.

The above mentioned experimental results motivated further experimental and theoretical studies on the diffusion of point defects at the GaN/AlN interfaces, performed in recent years. In Ref. [15], the authors studied the diffusion process at the GaN/AlN interfaces. In particular, in the framework of density functional theory, they calculated migration barriers and diffusion coefficient for Al and Ga atoms in bulk GaN and AlN crystals, as well as the Al–Ga interdiffusion coefficient in the case of vacancy mediated mechanism. They showed that Ga atoms exhibit higher diffusion coefficient in GaN and AlN than that of Al atoms. The reported experimental interdiffusion coefficient between GaN and AlN in the GaN/AN superlattice heterostructure was of the order of $10^{-20}$ ${\mathrm{cm}}^{2}$ $s^{-1}$ at 100 °C, in agreement with their theoretical calculations.

Next, Gao et al. [16], by means of ab initio calculations and four models of ${\mathrm{Al}}_{x}{\mathrm{Ga}}_{1-x}N/\mathrm{GaN}$ heterointerfaces with different Al content, studied the variations of band offsets (VBO) with interfacial structure, point defect position, as well as the electronic states of interfacial vacancies $V_{N}$, $V_{Ga}$ and anti–sites ($Ga_{N}$, $N_{Ga}$). The authors showed that the influence of $V_{N}$ and $Ga_{N}$ in the VBO was related to the defect location. $V_{N}$ present at the ${\mathrm{Al}}_{x}{\mathrm{Ga}}_{1-x}N$ side, in the second nitrogen layer next to the interface, shifted the VBO most significantly, while in the case of $Ga_{N}$ it was the one present in the topmost gallium layer at the interface. Gallium vacancy $V_{Ga}$ and N anti–site ($N_{Ga}$) modified the VBO consistently, regardless of their locations, and the anti–site influenced VBO much less than vacancy.

In a series of papers by Strak et al. [17,18,19] the issue of the presence of built-in and strain-induced electric fields in the multi-quantum well (MQWs) GaN/AlN systems was investigated both theoretically and experimentally. The authors investigated a relation between the electric field and the width of quantum wells and barriers, an influence of strain on the electric field values, as well as the optical transition energies in the GaN/AlN MQWs systems.

Undoubtedly, the problem of the formation and migration of defects is important for all applications of AlN/GaN heterostructures, where a high interface quality is required. The aim of the present paper is to find the heights of the migration energy barriers of all native point defects throughout the AlN/GaN interface, which were exempt from the analysis of papers [15,16]. In particular, neutral and charged nitrogen, gallium, and aluminium vacancies will be studied, as well as the complexes consisting of a substitutional metal atom and vacancy. Moreover, besides the most common vacancy mediated diffusion mechanism, two additional mechanisms will be discussed; i.e., the direct interstitial and indirect ones in bulk AlN and GaN crystals, as well at the AlN/GaN heterointerface. We will show that in the presence of charged point defects $V_{Ga}^{3-}$ and $V_{Al}^{3-}$ at the AlN/GaN heterointerface, not discussed by other authors, a new effect of the inversion of electric field can be observed, not reported so far. Our analysis is performed for two technologically important types of the AlN/GaN interfaces; the strained and relaxed ones, so as to take into account two possible growth modes of the AlN/GaN heterostructure, that is, the pseudomorphic growth of AlN on GaN substrate and the lattice-matched mode of AlN growth on GaN.

The present paper is organized as follows. Section 2 presents the details about the applied calculation method and model. Section 3 provides calculated heights of the migration energy barriers of native point defects in bulk GaN and AlN crystals for various diffusion mechanisms, while Section 4 presents the corresponding migration energy barriers encountered by point defects at the AlN/GaN heterointerface, together with the related profiles of electric fields. In all studied cases the preferred migration direction of defects are identified. Finally, Section 5 concludes the obtained results.

## 2. Models and Methods

Our calculations were performed based on the density functional theory (DFT) within the Kohn–Sham approach. We used Spanish Initiative for Electronic Simulations with Thousands of Atoms (SIESTA) software [20,21]. The exchange and correlation effects were taken into account by using the general gradient approximation (GGA) functional of Perdew, Burke and Ernzerhof with parameters β, μ fixed by the surface, jellium response, and κ fixed by the Lieb–Oxford bound for the low density limit of homogeneous electron gas (PBEJsJrHEG) [22,23,24]. The cutoff energy of 500 Ry was used to generate a real space mesh, and the Brillouin zone sampling was performed by means of a 15×15×9 Monkhorst–Pack *k*-points mesh for unit cell of bulk GaN and AlN crystals. Positions of all atoms in the studied systems were relaxed with the convergence criteria imposed on atomic forces to be less than 0.001 eV/Å in bulk nitride crystals, or 0.01 eV/Å in the AlN/GaN superlattice, respectively.

The chosen numerical parameters led to the lattice parameters and physical properties of both crystals, presented in Table 1.

The heights of the migration energy barriers in bulk wurtzite GaN and AlN crystals were computed using a 5×5×3 supercell model with 300 atoms. This size of the supercell, as shown in [25], ensured a necessary spatial separation between a defect and its periodic image, that is, ∼16.01 Å and 15.62 Å in the case of GaN and AlN supercells, respectively. For these systems, as well as for structures representing superlattices, the reciprocal space sampling was applied only at the Γ point.

**Table 1:** Calculated properties of AlN and GaN bulk crystal compared with relevant experimental data.

Property	AlN		GaN	
	This Paper	Exp.	This Paper	Exp.
Lattice constant *a* (Å)	3.124	3.111 [26]	3.215	3.190 [26]
Lattice constant *c* (Å)	5.013	4.981 [26]	5.220	5.190 [26]
Formation enthalpy $\Delta H^{f}$ (eV)	−3.32	−3.13 [26]	−1.34	−1.29, −1.34 [26,27]

**Figure 1:**
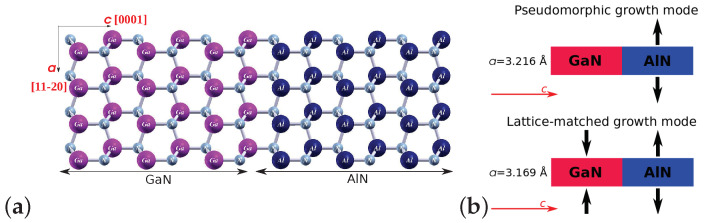
(**a**) The model of AlN/GaN superlattice, (**b**) schemes of the two growth modes of AlN/GaN heterointerface. Large violet and navy blue balls represent Ga and Al atoms, respectively, while small grey balls denote N atoms.

The AlN/GaN interface was represented by a stoichiometric superlattice model with a 4×4×6 size of unit cell, and the growth direction chosen parallel to the wurtzite *c* axis. The discussed model of superlattice is presented in Figure 1a and it consists of six double Ga(Al)–N layers, comprising 384 atoms. The presented AlN/GaN interface was additionally adapted to investigate an impact of in-plane strain on the defect migration by means of two interface types. In order to model a pseudomorphic growth of AlN on the GaN substrate, in the first type of heterostructure, *a* lattice parameter of AlN was fixed to that of GaN, whereas the *c* parameter was allowed to relax freely. Next, all atomic positions in the superlattice were optimized with the above mentioned restriction concerning forces. The obtained heterointerface was therefore strained to GaN (see Figure 1b upper panel), and the AlN part of heterostructure was subjected to a tensile strain. The second prepared model of the AlN/GaN heterointerface type represented a lattice-matched mode of AlN growth on GaN. In this model presented in the Figure 1b lower panel, the in-plane lattice parameters of the supercell were fixed to the average lattice constant of bulk GaN and AlN, that is, *a* = 3.169 Å, according to Vegard’s law. As before, positions of all atoms in the superlattice were fully relaxed, with the same restriction concerning forces. In this growth mode, the GaN substrate was subjected to a small compressive stress, while the AlN part of heterostructure was subjected to a tensile strain, smaller in value than that of the previous growth mode. It should be noted here that this model is a simplified one in which the analysis of the mismatch dislocation microstructure was not performed.

**Figure 2:**
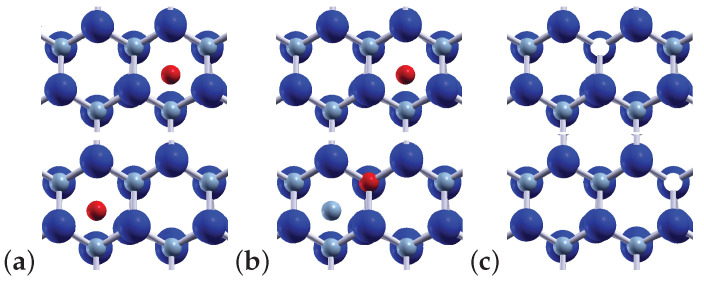
Diffusion mechanisms: (**a**) direct interstitial; (**b**) indirect interstitial; (**c**) vacancy mediated. Each top panel represents an initial site of defect migration, while the bottom one—a final site. Large navy blue balls represent Al atoms, small blue—N, red—interstitial N, and white—nitrogen vacancy $V_{N}$.

The heights of potential energy barriers related to point defects migration were calculated using three diffusion mechanisms, commonly discussed in the experiments, such as the direct interstitial, indirect interstitial and vacancy mediated ones [13,15]. The direct interstitial is the simplest diffusion mechanism, but it concerns only atoms that are considerably smaller than the host atoms. The geometry of the host lattice defines the interstitial sites and a defect can migrate by jumping from one site to the other one in its neighborhood (see Figure 2a) [28]. The next two diffusion mechanisms involve the motion of at least one or two atoms. In the case of the indirect interstitial diffusion mechanism, an extra atom located between lattice sites replaces a neighboring host atom, by forcing it to one of the neighboring interstitial sites (see Figure 2b). Finally, the vacancy mediated mechanism is a dominant one, and it is connected with atom jumps into a neighboring vacancy site (see Figure 2c). Such a jump of the atom is equivalent to the vacancy movement in the opposite direction; therefore, we will also use the notation about the diffusion of the vacancy. It should be noted here that the investigation of defect migration across the AlN/GaN interfaces was performed in recent papers only in the case of vacancy mediated mechanism [15,29].

In order to estimate the heights of the migration energy barriers of selected native point defects in our systems, we used the Nudged Elastic Band method (NEB) [30,31,32], according to our approach presented in Ref. [25], dedicated to interfaces between two materials.

## 3. Results

### 3.1. Heights of Migration Energy Barriers of Native Point Defects in Bulk GaN and AlN Crystals for Various Diffusion Mechanisms

In order to interpret data concerning the heights of energy barriers related to migration of points defects throughout the AlN/GaN interface, the information about the corresponding barrier’s heights in the case of bulk AlN and GaN crystals is necessary. Therefore, in the beginning, we calculated the migration energy barriers in bulk AlN and GaN crystals, taking into account the available data concerning possible charge states of selected point defects [33,34,35]. The heights of migration energy barriers were compared, whenever possible, with data from other papers. It should be noted here that an extensive analysis concerning defects in nitrides can be found in [36,37,38,39,40,41].

As can be seen from Table 2, the migration of aluminium vacancy $V_{Al}$ in bulk AlN is related to overcoming an energy barrier smaller by about ∼0.5 eV than that of nitrogen vacancy $V_{N}$. Such situation takes place both for the *a* and *c* directions. However, in the case of charged vacancies we observe a situation different to that of charged vacancies in bulk GaN [25]. The heights of energy barriers of $V_{N}^{3+}$ and $V_{Al}^{3-}$ in the lateral direction *a* are higher than those of $V_{N}^{0}$ and $V_{Al}^{0}$. Note that the notation $V_{N}^{3+}$, ($V_{N}^{3-}$) refers to the system in which three electron charges *e* are removed (added), so as it represents *p*-type doping (*n*-type doping). The analogous notation refers to charged metal vacancies and their complexes with substitutional atoms.

Since a diffusion of Al and Ga atoms is discussed in some experimental papers [13,15], we have calculated heights of energy barriers for complexes that consist of a substitutional Ga atom and a metal vacancy. Interestingly, the heights of migration energy barriers of both neutral and charged $Ga_{Al}+V_{Al}^{3-}$ complexes are smaller than that of a single $V_{Al}$ vacancy. This concerns both the migration along the *c* axis and the lateral *a*-axis direction. The largest lowering in height of the barrier of ∼0.45 eV is related to the migration of $Ga_{Al}+V_{Al}^{3-}$ along the *c* axis.

In the case of the indirect interstitial diffusion mechanism, the height of the energy barrier associated with the migration of Al interstitial atom along the *c* axis being smaller by about 0.8 eV than those of a single $V_{Al}$ vacancy and of the $Ga_{Al}+V_{Al}$ complex. As for the *a*-axis direction, this difference is less than 0.35 eV. In the case of the N interstitial atom, the migration energy barriers related to both diffusion mechanisms are similar in height: that is, the ∼1.8 eV (*c* direction) and the ∼2 eV (*a* direction). These values are still smaller than those of $V_{N}$, encountered at the vacancy mediated diffusion mechanism.

Table 3 presents calculated values of the energy barriers related to the migration of a substitutional Al atom creating a complex with gallium vacancy $Al_{Ga}+V_{Ga}$, as well as of interstitial $Ga_{i}$ and $N_{i}$ atoms, associated with two diffusion mechanisms in GaN crystal. The heights of energy barriers related to single $V_{Ga}$ and $V_{N}$ vacancies, as shown in our paper [25], were as follows: 2.75 eV and 4.06 eV, respectively. As follows from Table 3, an uncharged $Al_{Ga}+V_{Ga}$ complex migrates along the *c*-axis direction with the energy barrier larger by about 0.5 eV than that of the single gallium vacancy. However, in the case of the charged $Al_{Ga}+V_{Ga}^{3-}$ complex, an essential decrease in the height of the energy barrier in the *c*-axis direction is observed, down to 1.78 eV. This decrease is a result of the presence of spontaneous polarization along the *c* axis in wurtzite GaN crystal. Therefore, such a decrease is not observed in the lateral direction. It should be noted here that the calculated value 1.78 eV differs essentially from the value 2.58 eV reported by Alexandrov [15]. In our opinion, this difference is a consequence of a small supercell size used in [15], containing only 96 atoms (three times less than in our model). In the smaller computational model, there are fewer degrees of freedom and therefore the crystal lattice is stiffer and the calculated diffusion barriers may be higher.

A migration of a single interstitial $Ga_{i}$ metal atom along the *c*-axis direction is associated with the energy barrier of only 1.15 eV. Interestingly, the corresponding energy barrier in the *a* direction is even smaller and amounts to 0.51 eV. In the case of the interstitial $N_{i}$ atom, the height of the energy barrier related to its migration along the *c* axis is equal to 2.42 eV and this value is much smaller than that of nitrogen diffusion via the vacancy mediated mechanism (4.06 eV). Such a situation takes place for both diffusion mechanisms of nitrogen interstitial $N_{i}$. Similarly, it takes place for the $N_{i}$ during its migration along the lateral *a* direction.

### 3.2. Projected DOS Function of AlN/GaN Superlattice as a Function of the Atomic Positions

In the next step, we computed the spatial distribution of the density of states function projected on the atom quantum states (PDOS) both for the pseudomorphic and lattice-matched growth modes of the AlN/GaN heterointerface. In this part of the calculation, a correction $GGA-\frac{1}{2}$ was applied to improve the value of the energy bandgap of the studied system, that was underestimated in the standard GGA approach [43]. The energy bandgap of the bulk AlN with the account of $GGA-\frac{1}{2}$ was 5.90 eV, and that of GaN was 3.45 eV. The corresponding experimental values are as follows: $E_{g}^{AlN}=6.09$ eV [8] and $E_{g}^{GaN}=3.47$ eV [6,7].

**Figure 3:**
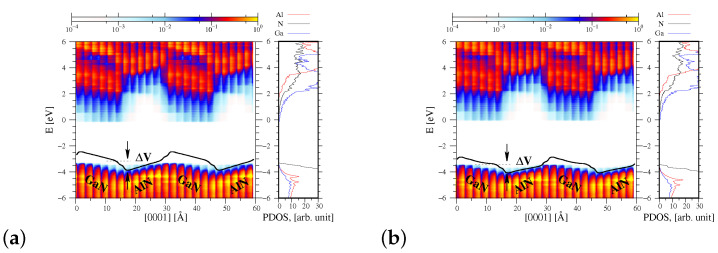
Energy band profiles for AlN/GaN superlattice: (**a**) strained to GaN, (**b**) fully relaxed. Left diagram—the density of states projected on the atom quantum states (PDOS), showing the spatial variation of the valence and conduction bands as a function of the AlN/GaN heterointerface height *z*, right diagram—PDOS associated with Al, N, and Ga atoms denoted by red, black, and blue lines, respectively. The potential jump at the interface are ΔV=0.76 eV and ΔV=0.67 eV for the strained (**a**) and relaxed (**b**) cases, respectively.

Figure 3a displays the PDOS function, as well as the associated profiles of the conduction and valence bands of the AlN/GaN heterojunction for the case when the AlN material is strained to GaN (pseudomorphic growth mode). A rapid change of the macroscopic average of the electrostatic potential denoted by a solid black line can be observed in Figure 3a, which indicates both the presence of the polarization charge, as well as of the dipole layers at the AlN/GaN interface area. This potential jump ΔV in the direction perpendicular to the interface is slightly smaller in the case of relaxed interface type (0.67 eV) than that of the strained one (0.76 eV) (compare Figure 3a,b).

## 4. Discussion

### 4.1. Diffusion of Point Defects across the AlN/GaN Strained Heterointerface Type

**Figure 4:**
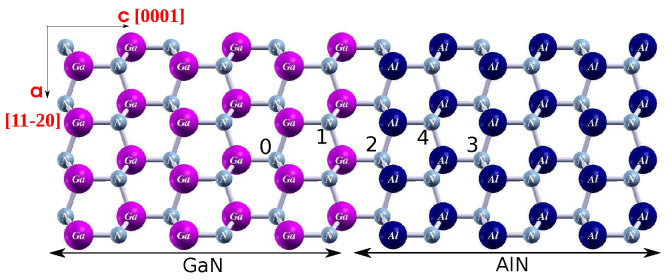
Model of the AlN/GaN interface with the studied lattice sites i = 0, ..., 4 of nitrogen vacancy $V_{N}^{0}$.

Next, we computed the joint profiles of energy barriers of the studied defects associated with their migration across the AlN/GaN interface, for both growth modes of the AlN/GaN heterostructure. Additionally, for each barrier’s profile we computed the corresponding shape of the electric field present at the AlN/GaN heterojunction. For this purpose, a macroscopic average of the course of electrostatic potential along the crystallographic *c* direction was found, and next, it was overlapped with the projected density of states function PDOS. A migration of defects was studied in the area next to the AlN/GaN heterointerface, between particular lattice sites related with defects. In the case of a nitrogen vacancy discussed below, four such exemplary sites are presented in Figure 4.

#### 4.1.1. Migration of $V_{N}^{0}$ and $V_{N}^{3+}$

**Figure 5:**
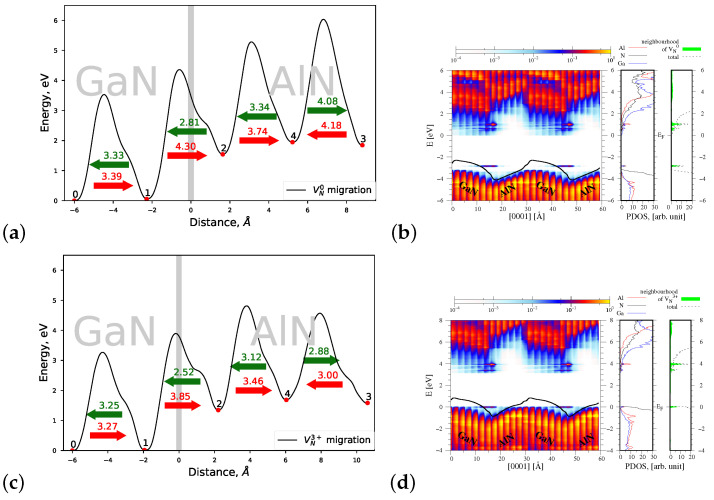
Left—joint profiles of the migration energy barriers of nitrogen vacancy $V_{N}$ at the AlN/GaN strained interface type, right—the density of states projected on the atom quantum states (PDOS), showing the spatial variation of the valence and conduction bands in the GaN/AlN heterostructure, as well as the total DOS and PDOS functions associated with Al, N, and Ga atoms, denoted by red, black, and blue lines, respectively. The green shade represents a sum of the PDOS resulting from the nearest neighbors of $V_{N}$. (**a**,**b**) $V_{N}^{0}$, the corresponding potential jump ΔV=0.97 eV (**c**,**d**) $V_{N}^{3+}$, the corresponding potential jump ΔV=0.74 eV.

Figure 5a shows a profile of the migration energy barriers of neutral nitrogen vacancy $V_{N}^{0}$ across the AlN/GaN strained heterointerface. The numbers 0–4 denote subsequent lattice sites in the interface area (compare Figure 4) where a formation of $V_{N}^{0}$ was taken into account. Point 0 corresponded to the most stable lattice site, where the formation energy of $V_{N}^{0}$ was minimal. The heights of energy barriers were always computed between the initial and final migration sites, for example, between 0 and 1, 1 and 2, and so on, according to the procedure described in detail in our previous paper [25]. Green arrows display the preferred motion direction of $V_{N}^{0}$, associated with overcoming a lower energy barrier pointed by the corresponding numbers. As can be seen in Figure 5a, $V_{N}^{0}$ migrates with a decreasing energy barrier from AlN towards GaN, to the lattice site 0, where its formation energy is the smallest. The lowest $E_{b}=2.81$ eV is needed to cross the interface. An increasing slope of the joint profile of energy barriers in the AlN part of heterointerface indicates that the migration of $V_{N}^{0}$ throughout AlN requires more and more energy. Moreover, it can be seen that the energy $E_{b}=2.81$ eV needed to overcome the interface in the direction inverse to the heterostructure growth is smaller both from the energy barriers of 3.50 eV and 4.06 eV, corresponding to the migration of $V_{N}^{0}$ in *c* direction in bulk AlN and GaN, respectively. The profile of the macroscopic average of the electrostatic potential computed along the heterostructure height *z* and overlapped with the PDOS function of the AlN/GaN in Figure 5b shows that some new energy states arise near the valence band top and the conduction band bottom, originating from Al and Ga atoms located in the neighborhood of $V_{N}^{0}$. These new energy states are presented additionally in the most right panel of Figure 5b as a PDOS function versus energy, in a very good agreement with the results of [16]. The potential jump ΔV caused by the presence of $V_{N}^{0}$ at the GaN/AlN strained interface type is increased as compared with the AlN/GaN heterointerface without defects and it amounts to ΔV=0.97 eV. This result is again comparable with that reported by Gao et al., ΔV=0.99 eV.

In the case of migration of a charged $V_{N}^{3+}$ vacancy, the corresponding profile of energy barriers presented in Figure 5c shows a small decrease in the heights of all barriers. The lowest migration energy barrier is again connected with overcoming the AlN/GaN interface between the lattice sites 2 and 1, and it amounts to 2.52 eV. The spatial distribution of the PDOS function, displayed in Figure 5d, presents a small redistribution of the nitrogen energy states that takes place near the Fermi energy level, coinciding with the valence band top of the system. As a consequence of the decrease in the barriers’ heights, the potential jump ΔV at the interface is also decreased to the value 0.74 eV.

#### 4.1.2. Migration of $V_{Al}^{0}$ and $V_{Al}^{3-}$

**Figure 6:**
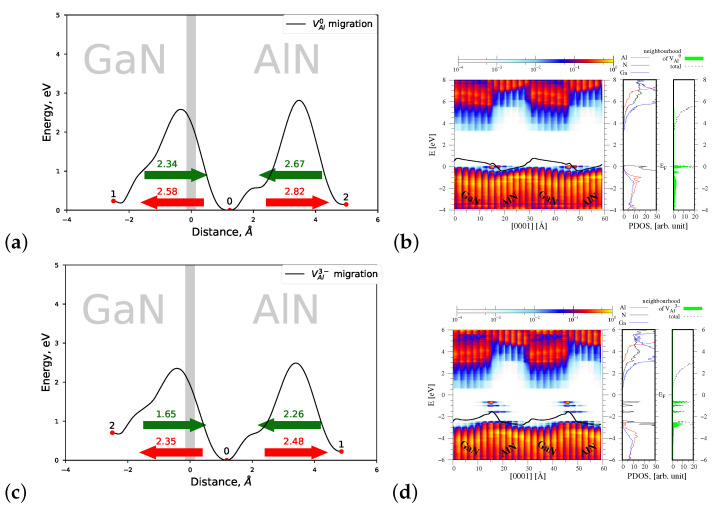
Left—joint profiles of the migration energy barriers of aluminium vacancy $V_{Al}$ at the AlN/GaN strained interface type, right—the density of states projected on the atom quantum states (PDOS), showing the spatial variation of the valence and conduction bands in the GaN/AlN heterostructure, as well as the total DOS and PDOS functions associated with Al, N, and Ga atoms, denoted by red, black, and blue lines, respectively. The green shade represents a sum of the PDOS from the nearest neighbors of $V_{Al}$. (**a**,**b**) $V_{Al}^{0}$, the corresponding potential jump ΔV=0.75 eV (**c**,**d**) $V_{Al}^{3-}$, the corresponding potential jump ΔV=0.58 eV.

Figure 6a displays a joint profile of the migration energy barriers of $V_{Al}^{0}$ vacancy across the AlN/GaN strained heterointerface. As follows from Figure 6a, a neutral $V_{Al}^{0}$ is created preferentially near the interface area in the AlN part of the heterostructure. Its possible migration occurs towards the interface, and it is associated with overcoming the energy barrier of $E_{b}=2.67$ eV. This value is smaller than the corresponding energy barrier of 2.97 eV, observed in a bulk AlN crystal in *c* direction. Note that the transition 1→0 connected with the minimal energy of 2.34 eV stands for a migration of *gallium vacancy* leading to the creation of aluminium vacancy in site 0 and of substitutional atom $Al_{Ga}$ in the lattice site 1. Again, as presented in Figure 6b, a number of new energy states appear near the valence band top, that originate this time from N atoms located in the neighborhood of $V_{Al}^{0}$.

In the case of charged $V_{Al}^{3-}$ vacancy, one observes further decrease in heights of energy barriers, connected with defect’s migration near the AlN/GaN heterointerface, without any change of the preferred direction of migration (see Figure 6c). However, the presence of charged $V_{Al}^{3-}$ vacancy changes essentially the spatial profile of the energy bands and of the resulting macroscopic average of the electrostatic potential, shown in Figure 6d, left panel. In our opinion, it corroborates the change of sign of the dipole layer, present in the interface area, and induced by the presence of charged $V_{Al}^{3-}$ vacancy. The change in profile of the energy bands is associated both with new energy states formed in the energy bandgap of heterostructure, mainly by nitrogen atoms located in the neighborhood of $V_{Al}^{3-}$, and with a small decrease in the potential jump value ΔV=0.58 eV, as compared with ΔV=0.75 eV of $V_{Al}^{0}$. The observed effect of the inversion of the electric field at the AlN/GaN heterointerface has not been reported so far. Hence, in the next section, it will be checked whether it can be related to the stress of the system.

#### 4.1.3. Migration of $V_{Ga}^{0}$ and $V_{Ga}^{3-}$

**Figure 7:**
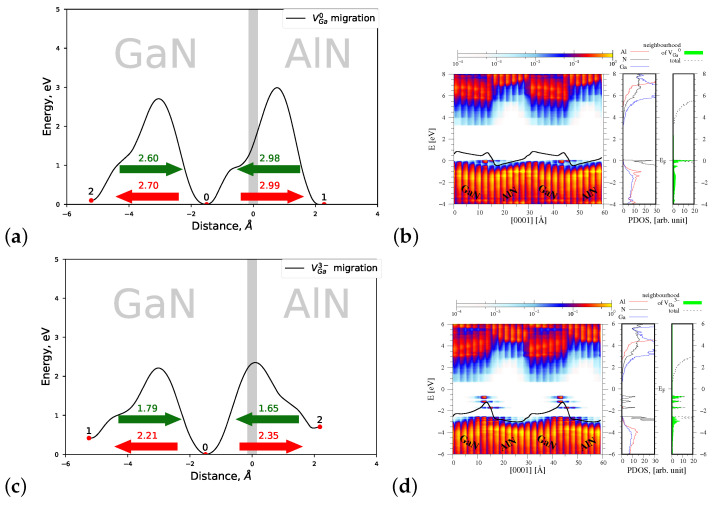
Left—joint profiles of the migration energy barrier of gallium vacancy $V_{Ga}$ at the AlN/GaN strained interface type, right—the density of states projected on the atom quantum states (PDOS), showing the spatial variation of the valence and conduction bands in the GaN/AlN heterostructure, as well as the total DOS and PDOS functions associated with Al, N, and Ga atoms, denoted by red, black, and blue lines, respectively. The green shade represents a sum of the PDOS from the nearest neighbors of $V_{Ga}$. (**a**,**b**) $V_{Ga}^{0}$, the corresponding potential jump ΔV=0.99 eV (**c**,**d**) $V_{Ga}^{3-}$, the corresponding potential jump ΔV=1.35 eV.

An uncharged gallium vacancy $V_{Ga}^{0}$ is created with the smallest formation energy in the interface area, in the GaN part of the heterostructure (see Figure 7a). Its preferred migration takes place from the deeper GaN layers towards the interface. The energy necessary to reach the interface amounts 2.60 eV, and this value is again smaller than the migration energy barrier $E_{b}=2.75$ eV of $V_{Ga}^{0}$ in bulk GaN, in *c* direction. In the case of charged $V_{Ga}^{3-}$, its migration also occurs from the deeper GaN layers towards the interface AlN/GaN, and the energy necessary to arrive at the interface $E_{b}=1.79$ eV is essentially less than that of $V_{Ga}^{0}$. Similarly to the charged aluminium vacancy $V_{Al}^{3-}$, the other energy barrier related to the migration of $V_{Ga}^{3-}$ in the AlN part of heterostructure is also decreased. Hence, it can be stated that both metal vacancies exhibit a preferred migration direction towards the interface, nonetheless, such migration takes place from the respective host material.

Figure 7b (left panel) presents a spatial distribution of the PDOS function and of the energy bands overlapped with the macroscopic average of the electrostatic potential, denoted by a solid black line. As can be observed, a potential jump ΔV=0.99 eV at the AlN/GaN interface caused by the presence of $V_{Ga}^{0}$ is larger than that of induced by $V_{Al}^{0}$ (ΔV=0.75 eV, compare Figure 6b). In analogy to the situation observed for the charged aluminium vacancy, the presence of $V_{Ga}^{3-}$ in the interface area changes considerably the profile of energy bands with respect to that of $V_{Ga}^{0}$. The potential jump ΔV at the interface is equal to 1.35 eV. The change of the profile of energy bands corroborates the change in sign of the dipole layer present at the interface with respect to that of $V_{Ga}^{0}$. Hence, it can be stated that both charged metal vacancies $V_{Ga}^{3-}$ and $V_{Al}^{3-}$ change the profile of energy bands near the AlN/GaN interface area.

Additionally, new energy states can be observed in the energy gap of the AlN/GaN heterostructure (compare Figure 7d right panel), which originate mainly from the nitrogen atoms situated in the neighborhood of $V_{Ga}^{3-}$. The last observation is again in a very good agreement with data reported by Gao [16].

#### 4.1.4. Migrations of $N_{i}$ and $Me_{i}$ Atoms via the Indirect Diffusion Mechanism

**Figure 8:**
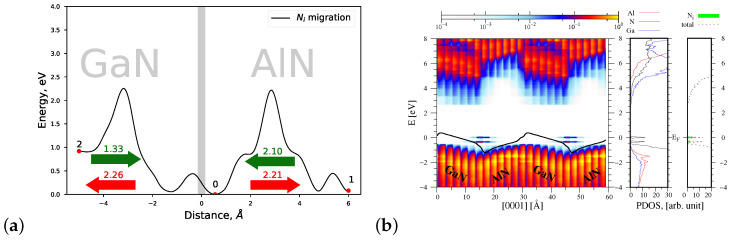
(**a**) Joint profile of the migration energy barrier of nitrogen interstitial $N_{i}$ at the AlN/GaN strained interface type, (**b**) the density of states projected on the atom quantum states (PDOS), showing the spatial variation of the valence and conduction bands in the GaN/AlN heterostructure, as well as the total DOS and PDOS functions associated with Al, N and Ga atoms, denoted by red, black, and blue lines, respectively. The green shade represents PDOS resulting from $N_{i}$ interstitial.

Finally we computed the heights of energy barriers related to the migration of nitrogen and metal interstitial atoms across the strained AlN/GaN interface type. As can be observed in Figure 8a, $N_{i}$ can be created with the minimal formation energy in the AlN part of the AlN/GaN heterointerface area. Its possible migration occurs towards the interface, from the deeper interstitial sites of GaN and AlN materials; moreover, the lowest migration energy barrier $E_{b}=1.33$ eV is encountered when $N_{i}$ approaches the interface from the GaN substrate’s side. The obtained height $E_{b}=1.33$ eV of the energy barrier is the lowest amongst all barriers overcome by point defects during their migration near the AlN/GaN interface; moreover, it is essentially less than that of $N_{i}$ migrating in the *c* direction in bulk GaN (2.42 eV). Note also that the joint profile of the migration energy barriers of $N_{i}$ exhibits two smaller barriers of the height of 0.5 eV. They are connected with a rotational behavior of $N_{i}$ that takes place during the migration process both in GaN and AlN materials. Such behavior was revealed in bulk GaN in studies on the dynamics of $N_{i}$ interstitials [41].

The corresponding profile of the energy bands of the AlN/GaN heterostructure with one $N_{i}$ interstitial atom is similar to that of $V_{N}$ (see Figure 8b). The only difference is that new energy states originating from $N_{i}$ are created near the top of the valence band.

**Figure 9:**
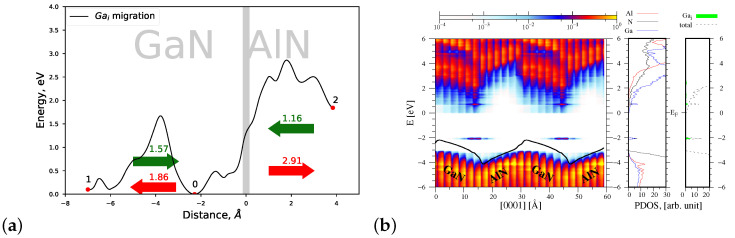
(**a**) Joint profile of the migration energy barrier of gallium interstitial $Ga_{i}$ at the AlN/GaN strained interface type, (**b**) the density of states projected on the atom quantum states (PDOS), showing the spatial variation of the valence and conduction bands in the GaN/AlN heterostructure, as well as the total DOS and PDOS functions associated with Al, N, and Ga atoms, denoted by red, black and blue lines, respectively. The green shade represents PDOS resulting from $Ga_{i}$ interstitial.

The migration of interstitial metal atoms $Al_{i}$ and $Ga_{i}$ near the AlN/GaN interface area proceeds in a similar way, and it will be illustrated in the example of $Ga_{i}$ (see Figure 9a). Both interstitial atoms exhibit the lowest formation energies at the GaN’s interface side. $Ga_{i}$ is created with the minimal formation energy far away from the interface (beneath 2nd Ga monolayer). When $Ga_{i}$ migrates from the GaN substrate towards AlN, its migration requires overcoming increasing potential barriers. The lowest barrier, about ∼1.6 eV, is still larger than that in a bulk GaN material (1.15 eV), in *c* axis direction, while the barrier related to crossing the interface $E_{b}=2.8$ eV is comparable with that of gallium vacancy.

The corresponding profile of energy bands is presented in Figure 9b. As can be seen, both the spatial profile of bands and the course of the average electrostatic potential, marked by a solid black line, are analogous to those of Figure 3a. It follows from the PDOS function presented in Figure 9b right panel that the energy states of $Ga_{i}$ are created in the energy gap, above the valence band top of the system.

### 4.2. Diffusion of Point Defects across the AlN/GaN Relaxed Interface Type

#### 4.2.1. Migration of $V_{N}^{0}$ and $V_{N}^{3+}$

**Figure 10:**
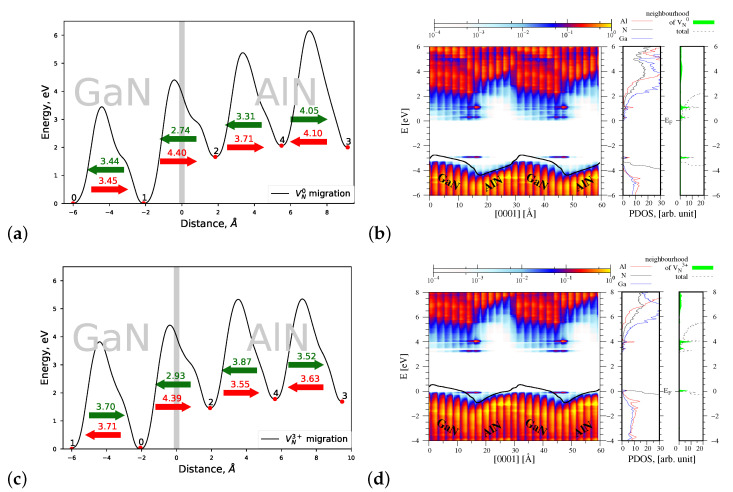
Left—joint profiles of the migration energy barrier of nitrogen vacancy $V_{N}$ at the AlN/GaN interface, right—the density of states projected on the atom quantum states (P-DOS), showing the spatial variation of the valence and conduction bands in the GaN/AlN and the total DOS is denoted by black dashed line; DOS associated with Al, N, and Ga atoms are denoted by red, black, and blue line, respectively; The green shade represents sum of the PDOS from the nearest neighbors of $V_{N}$. (**a**,**b**) $V_{N}^{0}$, the corresponding potential jump ΔV=0.90 eV (**c**,**d**) $V_{N}^{3+}$, the corresponding potential jump ΔV=0.66 eV.

The joint profiles of the migration energy barriers related to the migration of vacancies across the AlN/GaN heterointerface were also computed for the second growth mode of AlN/GaN heterointerface, that is, the lattice-matched one. As was mentioned before, this growth mode resulted in the relaxed AlN/GaN interface type, and the GaN substrate was subjected to a small compressive strain in the direction perpendicular to the heterostructure growth, while the AlN part was subjected to a small tensile strain in the same direction. Since the average lattice constant *a* utilized in this case is closer to that of relaxed bulk AlN, one should expect a smaller increase in the heights of energy barriers, connected with the migration of defects in the GaN part of the studied heterostructure. Figure 10a presents a joint profile of the migration energy barrier of neutral vacancy $V_{N}^{0}$ throughout the AlN/GaN interface. As expected, as compared to the strained heterointerface type, the height of the migration energy barrier of $V_{N}^{0}$ in the GaN part is slightly increased, while the heights of barriers in the AlN part are slightly decreased. The energy $E_{b}=2.74$ eV is needed for $V_{N}^{0}$ to cross the interface. Figure 10b shows the spatial distribution of the valence and conduction bands, as well as of the electric field profile in the interface area. The only difference that can be found in the course of the electric field as compared to that of the strained heterointerface type is its smoother slope and, correspondingly, a smaller potential jump ΔV=0.90 eV. The spatial distribution of energy states near the valence band top and the conduction band bottom, induced by the presence of $V_{N}^{0}$, is practically the same as in the case of strained AlN/GaN heterointerface type (compare Figure 6b and Figure 10b).

When a charged vacancy $V_{N}^{3+}$ is created in the interface area, then similarly, as is observed for the pseudomorphic growth mode, $V_{N}^{3+}$ migrates from the AlN part towards the GaN substrate, overcoming decreasing energy barriers. The energy required to cross the interface is the lowest amongst all barriers and it amounts to 2.93 eV. As compared to the strained heterointerface type, all migration energy barriers are increased by about 0.4–0.7 eV (compare Figure 5c and Figure 10c). The preferred migration direction remains the same, and the spatial distribution of energy bands and of PDOS function is practically the same as in the case of $V_{N}^{3+}$ created in the pseudomorphic growth mode of the AlN/GaN heterointerface (compare Figure 6d and Figure 10d).

#### 4.2.2. Migration of $V_{Al}^{0}$ and $V_{Al}^{3-}$

**Figure 11:**
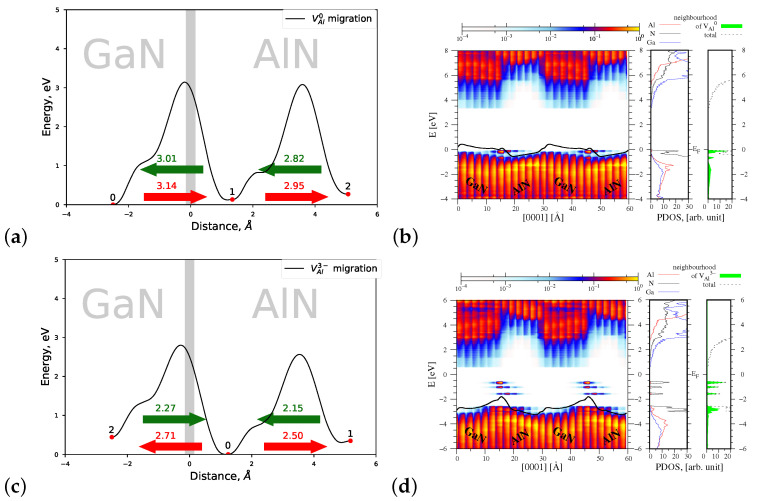
Left—joint profiles of the migration energy barrier of aluminium vacancy $V_{Al}$ at the AlN/GaN interface, right—the density of states projected on the atom quantum states (P-DOS), showing the spatial variation of the valence and conduction bands in the GaN/AlN and the total DOS is denoted by black dashed line; DOS associated with Al, N, and Ga atoms are denoted by red, black, and blue line, respectively; The green shade represents sum of the PDOS from the nearest neighbors of $V_{Al}$. (**a**,**b**) $V_{Al}^{0}$, the corresponding potential jump ΔV=0.61 eV (**c**,**d**) $V_{Al}^{3-}$, the corresponding potential jump ΔV=0.56 eV.

The migration energy barriers of $V_{Al}^{0}$ are presented in Figure 11a. Their heights are increased as compared to those of the strained AlN/GaN heterointerface type. The increase in heights is maximally up to 0.6 eV in the GaN part, and up to 0.15 eV in the AlN part. The lowest energy barrier is now 2.82 eV, while the preferred migration direction remains unchanged. In the case of $V_{Al}^{3-}$, as compared to the strained heterointerface type, a migration energy barrier related to the preferred motion direction is decreased down to 2.15 eV (by ∼0.1 eV) (see Figure 11c). This behavior can be explained by the fact that this time the average lattice constant *a* of the relaxed heterointerface type is closer to that of the bulk AlN. The spatial distribution of the energy bands and PDOS function are very similar to those of $V_{Al}$ in the strained AlN/GaN heterointerface type and the effect of the inversion of the electric field is still observed when a charged $V_{Al}^{3-}$ is present in the interface area.

#### 4.2.3. Migration of $V_{Ga}^{0}$ and $V_{Ga}^{3-}$

**Figure 12:**
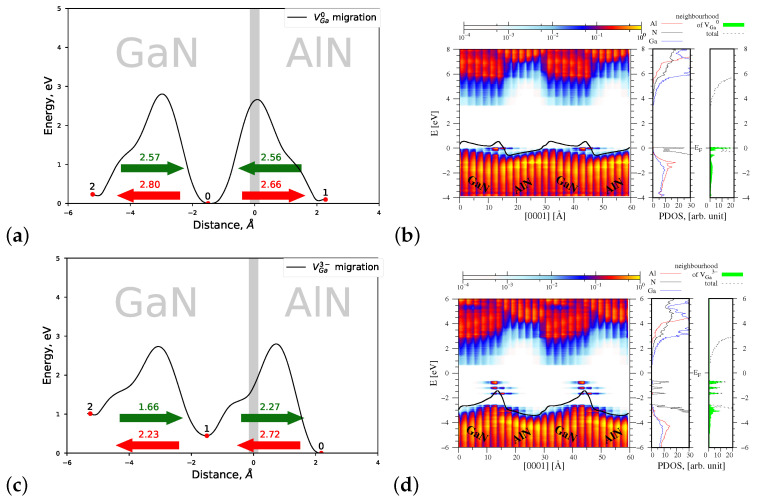
Left—joint profiles of the migration energy barrier of gallium vacancy $V_{Ga}$ at the AlN/GaN interface, right—the density of states projected on the atom quantum states (P-DOS), showing the spatial variation of the valence and conduction bands in the GaN/AlN and the total DOS is denoted by black dashed line; DOS associated with Al, N, and Ga atoms are denoted by red, black, and blue line, respectively; The green shade represents sum of the PDOS from the nearest neighbors of $V_{Ga}$. (**a**,**b**) $V_{Ga}^{0}$, the corresponding potential jump ΔV=0.85 eV (**c**,**d**) $V_{Ga}^{3-}$, the corresponding potential jump ΔV=1.32 eV.

Figure 12a,c present profiles of the energy barriers connected with the migration of $V_{Ga}^{0}$ and $V_{Ga}^{3-}$ in the relaxed heterointerface type. By comparing Figure 7a,c and Figure 12a,c, it can be stated that the preferred motion direction of both defects practically is not changed; it occurs from the deeper layers in GaN towards the interface. The lowest energy barrier is observed for $V_{Ga}^{3-}$ and it amounts to 1.66 eV. This value is essentially less than $E_{b}=2.28$ eV, related to the migration of $V_{Ga}^{3-}$ in the *c* direction in bulk GaN crystal [25]. Similarly to the situation discussed for the charged $V_{Al}^{3-}$ vacancy, an inversion of the slope of the macroscopic average of the electrostatic potential with respect to that of $V_{Ga}^{0}$ is again observed. This effect is induced by the presence of charged metal vacancies $V_{Ga}^{3-}$ or $V_{Al}^{3-}$ at the AlN/GaN interface area, and our investigation shows that the strain present in the system has no impact on this effect.

## 5. Conclusions

A diffusion of the native point defects throughout the AlN/GaN heterointerface was examined in the present paper. In particular, using DFT, we computed the heights of the migration energy barriers of neutral and charged nitrogen, gallium, and aluminium vacancies, as well as their complexes with the substitutional III-group element. Three diffusion mechanisms, that is, the vacancy mediated, direct interstitial, and indirect ones in bulk AlN and GaN crystals, as well as at the AlN/GaN heterointerface, were taken into account. Moreover, two growth modes of the AlN/GaN heterostructure along the hexagonal *c* direction were examined; the pseudomorphic and lattice-matched ones.

Our studies showed that the preferred migration direction of $V_{N}$ took place from the AlN material towards GaN with the lowest energy of 2.81 eV, needed to cross the interface between these two materials. Charged nitrogen vacancies $V_{N}^{3+}$ exhibited the same behavior and their motion was accompanied by a small decrease in heights of all barriers. Both metal vacancies exhibited the same preferred migration direction, that is, towards the AlN/GaN interface, and this migration took place from the respective host material. Therefore, their accumulation could be observed either at the AlN part of the interface (in the case of $V_{Al}$) or at the GaN part ($V_{Ga}$), independently of their charge state. The corresponding heights of the migration energy barriers of both neutral metal vacancies were slightly lower than that of nitrogen vacancy, of the order of ∼2.60 eV. In the case of charged metal vacancies, the heights of all migration barriers were decreased, down to ∼1.80 eV, in the case of $V_{Ga}^{3-}$. An interesting behavior related to the profile of energy bands of the AlN/GaN system was observed, namely an inversion of the profile of the macroscopic average of the electrostatic potential in the presence of charged $V_{Al}^{3-}$ and $V_{Ga}^{3-}$ vacancies at the interface, with respect to that induced by neutral metal vacancies. It was interpreted to be a result of the change of sign of the dipole layer, present at the interface area. This phenomenon should be elaborated further; at this stage of research, it could be stated that the strain present in the system had no impact on this effect.

Finally, we showed that, in general, all the studied migration energy barriers of gallium vacancy $V_{Ga}$ were slightly decreased in heights in the case of the lattice-matched growth mode of the AlN/GaN heterostructure.

## Figures and Tables

**Table 2:** Heights of the migration energy barriers in bulk wz-AlN. A positive charge state of the vacancy represents the *p*-type dopant, while a negative one, the *n*-type dopant. Explanation of symbols: $V_{N}$—nitrogen vacancy, $V_{Al}$—aluminium vacancy, $Ga_{Al}+V_{Al}$—substitutional Ga atom in Al sublattice migrating towards aluminium vacancy, $Al_{i}$—aluminium interstitial, $N_{i}$—nitrogen interstitial.

Defect/Mechanism	Charge State	Diffusion Direction	Energy Barrier $E^{b}$, eV	
			This Paper	Other Papers
$V_{N}$	0	*c*	3.50	3.37 [35]
		*a*	2.78	
	3+	*c*	3.00	
		*a*	3.02	
$V_{Al}$	0	*c*	2.97	
		*a*	2.37	
	3−	*c*	2.36	2.33 [15]
		*a*	2.88	
${\mathrm{Ga}}_{Al}$+$V_{Al}$	0	*c*	2.52	
		*a*	2.28	
	3−	*c*	1.90	1.74 [15]
		*a*	2.05	
${\mathrm{Al}}_{i}$ indirect	0	*c*	1.72	
		*a*	2.02	
$N_{i}$ indirect	0	*c*	1.77	
		*a*	2.12	
$N_{i}$ direct	0	*c*	1.89	
		*a*	1.91	1.24 [35]

**Table 3:** Heights of the migration energy barriers in bulk wz-GaN. A positive charge state of the vacancy represents *p*-type dopant, while a negative one, *n*-type dopant. Explanation of symbols: $Al_{Ga}+V_{Ga}$—substitutional Al atom in Ga sublattice migrating towards gallium vacancy, $Ga_{i}$—gallium interstitial, $N_{i}$—nitrogen interstitial.

Defect/Mechanism	Charge State	Diffusion Direction	Energy Barrier $E^{b}$, eV	
			This Paper	Other Papers
${\mathrm{Al}}_{Ga}$+$V_{Ga}$	0	*c*	3.24	
		*a*	2.54	
	3−	*c*	1.78	2.58 [15]
		*a*	2.11	
${\mathrm{Ga}}_{i}$ indirect	0	*c*	1.15	
		*a*	0.51	
$N_{i}$ indirect	0	*c*	2.42	
		*a*	2.03	
$N_{i}$ direct	0	*c*	2.42	2.4 [38,41,42]
		*a*	2.33	2.33 [42], 2.34 [41], 2.4 [35,38]

## Data Availability

The data presented in this study are available on request from the corresponding author.
